# Therapeutical and Pharmaceutical Notes

**Published:** 1900-12

**Authors:** W. J. Martin

**Affiliations:** Kankakee, Ill.


### THERAPEUTICAL AND PHARMACEUTICAL NOTES.

UNDER THE DIRECTION OF W. J. MARTIN, M.D.C.,
KANKAKEE, ILL.

GASOLINE, OR PETROLEUM ETHER, AS A SURGICAL DETERGENT. In a paper read before the International Association of Railway Surgeons at Detroit, Dr. B. L. Riordan, of Toronto, unreservedly advocated the use of ordinary gasoline as the best agent for preparing the field of operation. He was first led to try this agent on a railway employé whose crushed hand was grimy with the black oil of the mechanic, and has been using it for four years with the best of satisfaction. He finds that it does not irritate fresh wounds or granulating surfaces any more than does water. It is best applied by taking an ordinary wire made of cotton-batting or sterilized gauze and wiping the parts which it is desired to cleanse. The gasoline immediately evaporates and leaves the surface dry and clean and perfectly free from grease. This will be found an advantage where sectional strapping by adhesive plaster is to be used, as the plaster adheres much more firmly when the skin is free from any oily substance. His results, as far as early healing of and absence of infection are concerned, have been most satisfactory, and include the treatment of all classes of wounds. Of course, the inflammable nature of the gasoline must not be forgotten.

A DECINORMAL SALT SOLUTION. A decinormal salt solution is thus explained in the *Medical Record:* A normal solution in volumetric chemistry is a solution containing in 1000 c.c. an amount of the active constituent just sufficient to combine with or replace 1 gramme of hydrogen. In the case of sodium chloride this amount is 58.37 grammes; hence a normal salt solution is one containing 58.37 grammes to the litre of water. A solution one-tenth of this strength (a decinormal solution) contains 5.837 grammes to the litre of water, which is just about the strength of the saline solution used for venous infusion.

EPICARIN. Epicarin is a condensation product of cresotin acid and betanaphthol, and represents an acid which is capable of forming easily soluble neutral salts, differing in this respect from betanaphthol, which forms only alkaline (caustic) phenolates. This property serves to explain the comparative innocuousness of epicarin, since, according to Dresse, a fish when placed in a $\frac{1}{16}$ per cent. solution of betanaphthol became motionless in twenty seconds, whereas this effect developed only after three minutes in a solution of epicarin of $\frac{1}{4}$ per cent., viz., four times as strong. Doses of 0.2 gramme per os were well tolerated by rabbits without the occurrence of general symptoms of poisoning or the presence of albumin in the urine. The greater portion of the product could be recovered from the urine by shaking with ether. As regards bacteriological examination, experiments in Dresse's laboratory showed an almost complete cessation of yeast fermentation and a destruction of the staphylococcus after an hour and a half. Epicarin has been used with good results in chronic eczema, scabies, prurigo, herpes tonsurans, etc.

Treatment of Equine Mange. Of the various forms of mange I will simply state that they have been prevalent in Edinburgh for some time. In many cases both the sarcoptic and psoroptic mites have been found. The symbiotic, affecting the legs, is always with us.

We found a very simple treatment to be very successful. Dress the body all over with any oil, train-oil, forty parts ; sulphur, one part. Wash off at the end of a fortnight, and dress again. Very few cases require a third dressing, and in three weeks they were fit to be discharged. By watching carefully for the slightest symptom of itchiness, isolating the horse, dressing him, and thoroughly washing the harness with water, soap, and Jeyes fluid (crude creolin), we were enabled to prevent the spread of the disease in a very large stud. — Professor W. Williams, in the *Veterinary Journal.*

Urticaria. The next skin disease to which I wish to refer is "urticaria," surfeit, or nettlerash. Like many other diseases, this is not so common now as it used to be, due undoubtedly to the more careful feeding and management of horses. The symptoms are manifested very suddenly. There may or may not be a preceding pruritus ; and the lesions characteristic of the disease are elevations of the skin, exactly resembling those produced by the sting of the nettle (urtica, a nettle). Now this disease is due to reflex irritation, arising from some disturbance of the digestive tract, and the formation of some excretory product, some say excess of urea.

In many instances the symptoms disappear very quickly, but I have seen cases most obstinate in their nature.

*Treatment.* Aperients, some soothing dressing to the skin, and in my experience an ounce of Scheele's prussic acid in about two quarts of water applied to the skin gives rapid relief. In chronic cases the origin must be carefully considered. Arsenic is recommended by some after aperients. I must say that I have been greatly disappointed with arsenic, and have substituted iod. pot. and colchicum, or, if there be debility, iron, bitters, and colchicum.—*Ibid.*

Immunity of Lower Animals. Prof. Metchnikoff (*Lancet*) has recently shown that the crocodile is almost entirely proof against tetanus. It is also known that in the crocodile it is impossible to produce a rise of temperature. The curious fact is also pointed out that mushrooms and moles, as well as bacteria, act upon toxins, converting them into vaccines, and without producing antitoxin. The invertebrates do not produce antitoxins to any extent.

Perfect health is that condition of the body when digestion is so perfect that the physiological balance between the destruction and construction that goes on ceaselessly in the cell-life is daily kept normal.

Detection of Salicylic Acid in Milk. Since it has lately been pointed out by Langkopf that the presence of citric acid interferes with the color reaction between salicylic acid and ferric chloride, Suess (*Pharm. Centralb.*) has investigated how far this is true in the case of milk, which naturally contains 0.17 to 0.2 per cent. of citric acid. He finds that the color reaction is visible when the clear serum of the milk in question is caused to drop through a layer of ether 50 c.c. in thickness, allowing the ether, after separation, to evaporate, and then adding to the residue one or two drops of dilute ferric chloride solution. In this manner he has clearly detected as little as 0.005 gramme of salicylic acid in 100 c.c. of milk.

Sodium Bifluoride for Histological Preparations. Marpmann (*Phar. Zeit.*) finds sodium bifluoride ($HNaF_{12}$) excellent for preserving histological, microscopical, or botanical specimens, preferring it to formaldehyde or mercuric chloride, because not poisonous in dilute solutions. It is without effect upon gelatin and the colors of plants. The author employs the following solution for "fixing" the lower organisms: Sodium fluoride, 0.5; sodium bifluoride, 2.0; water, 100. For animal tissue a 5 per cent. solution of sodium bifluoride is recommended. Snakes, etc., preserved their various variegated colors very well in the following solution: Sodium bifluoride, 5; glycerin, 50; alcohol, 100; water, 400. For preserving the organs a 10 per cent. aqueous solution of sodium chloride which contains 1.65 per cent. of sodium fluoride is recommended. Glands and brain substances are hardened by immersion for three or four days in a mixture of equal parts of Müller's fluid and water which contains 5 per cent. of sodium fluoride and 5 per cent. of a 40 per cent. formaldehyde solution.

HÆMOSTATIC ACTION OF GELATIN. According to the *New York Medical Times* favorable results have been obtained in inoperable cases of aneurisms by the injection of gelatin. The operation or treatment is not devoid of risk. A 5 per cent. or a 10 per cent. solution of dry gelatin in distilled water, to which is added 2 per cent. of chloride of calcium, has a remarkable hæmostatic action when applied locally.

DANGER IN SPINAL ANÆSTHESIA. Already reports of sudden deaths following this new operation are beginning to appear in the columns of the medical press. Gumprecht reports two cases of sudden death in his own practice, and refers to fifteen other cases where death occurred suddenly following the production of spinal anæsthesia.

HYPODERMATIC INJECTION OF COCAINE quickly relieves the intense pain of bee and wasp stings.

IT is said that permanganate of potash in the proportion of half a grain to a quart of water will render nux vomica (also strychnine) taken in poisonous doses a harmless compound.

IF there is not sufficient time to disinfect the operative field in the proper manner a thorough soaking with a 1 : 200 solution of mercuric chloride will often answer the purpose.

